# Case Report: A novel approach to prevent chronic histiocytic intervillositis and recurrent pregnancy loss by targeting maternal alloimmunity

**DOI:** 10.3389/fimmu.2025.1693016

**Published:** 2025-11-10

**Authors:** Mathilde Gavillet, Carole Gengler, Helene Legardeur, Monique Gannagé, Jardena Puder, Lydie Beauport, Alice Panchaud, Samuel Rotman, Denis Comte, David Baud, Dela Golshayan

**Affiliations:** 1 Service of Hematology, Department of Oncology, Lausanne University Hospital, Lausanne, Switzerland; 2 Central Laboratory of Hematology, Department of Laboratory Medicine and Pathology, Lausanne University Hospital, Lausanne, Switzerland; 3 Institute of Pathology, Department of Laboratory Medicine and Pathology, Lausanne University Hospital and University of Lausanne, Lausanne, Switzerland; 4 Woman-Mother-Child Department, Lausanne University Hospital and University of Lausanne, Lausanne, Switzerland; 5 Service of Immunology and Allergy, Lausanne University Hospital, Lausanne, Switzerland; 6 Service of Pharmacy, Lausanne University Hospital and University of Lausanne, Lausanne, Switzerland; 7 Institute of Primary Health Care (BIHAM), University of Bern, Bern, Switzerland; 8 Division of Internal Medicine, Department of Medicine, Lausanne University Hospital and University of Lausanne, Lausanne, Switzerland; 9 Transplantation Centre and Transplantation Immunopathology Laboratory, Department of Medicine, Lausanne University Hospital and University of Lausanne, Lausanne, Switzerland

**Keywords:** pregnancy, chronic histiocytic intervillositis, CIUE, allograft, immunosuppressive therapy, pregnancy loss, live birth

## Abstract

Recurrent miscarriage is a distressing condition with limited therapeutic options. Chronic histiocytic intervillositis of unknown etiology (CIUE) is a rare inflammatory placental disorder characterized by maternal immune cell infiltration of the intervillous space, fibrin deposition, and ischemic tissue damage, leading to pregnancy loss. The condition likely reflects an immune response against paternal alloantigens, with histopathological features resembling antibody-mediated rejection in solid organ transplantation. We investigated two women with recurrent CIUE-related pregnancy losses. Detailed immunological profiling included anti-human leukocyte antigen (HLA) antibody characterization, compatibility testing, and histopathological examination of previous placentas, as well as screening for other causes of recurrent pregnancy losses. Based on evidence of antibody-mediated alloimmune injury, we implemented a targeted immunosuppressive regimen derived from transplantation medicine, combining intravenous immunoglobulins (IVIG), tacrolimus, corticosteroids, and hydroxychloroquine, with close pregnancy monitoring. The first patient, after six consecutive CIUE-related pregnancy losses, underwent preconception desensitization and continued treatment throughout pregnancy. Early signs of placental dysfunction prompted therapy intensification, leading to delivery of a viable infant at 33 + 2 weeks. Placental histology showed only minor residual CIUE lesions. The second patient, with two pregnancy losses and a fetal demise from CIUE, began treatment at 6 weeks’ gestation and delivered a healthy infant at 36 weeks. In both cases, therapy was generally well tolerated, with gestational diabetes as the main complication, and no major maternal or neonatal adverse events. These cases support the concept that CIUE represents a breakdown of maternal immune tolerance toward paternal antigens, mediated by fetal-specific anti-HLA antibodies—akin to solid organ graft rejection. An immunosuppressive protocol adapted from transplantation medicine achieved two successful live births after multiple CIUE-related pregnancy losses. Targeting antibody-mediated alloimmunity may represent a promising therapeutic strategy for selected patients with recurrent miscarriages due to CIUE. Further studies are warranted to define optimal regimens and identify predictors of response.

## Introduction

Approximately 25% of couples will experience at least one pregnancy loss during their life, typically occurring in the first trimester of pregnancy (1). Recurrent pregnancy losses (RPL), defined as the loss of two or more pregnancies, impact about 3% of individuals, with extensive investigations identifying a cause in only half of them (2, 3). The maternal risk factors include coagulation disorders, uterine anomalies, hormonal imbalance, infections, and immunological disorders, in addition to chromosomal abnormalities of both parents.

Chronic inflammatory placental disorders are defined histopathologically by characteristic inflammatory infiltrates at the maternal-fetal interface leading to disrupted exchanges, after exclusion of infectious etiologies (4). One such disorder, chronic histiocytic intervillositis of unknown etiology (CIUE), is characterized by fibrin deposition and the infiltration of maternal CD68^+^ histiocytes within the intervillous space, i.e. the maternal interface (4). This condition frequently results in early (first trimester) pregnancy loss. Recurrent CIUE has been associated with autoimmunity, but is more likely driven by abnormal alloimmune responses, as severity increases with subsequent pregnancies (recurrence 67%) and re-exposure to identical paternal human leucocyte antigens (HLA) (4, 5).

Pregnancy typically represents a state of immunological tolerance toward the semi-allogeneic fetus, through the upregulation of immunoregulatory cells and soluble mediators in the placenta and maternal peripheral blood (6, 7). The invading fetal trophoblastic cells do not express immunogenic HLA antigens, but only the non-classical class I HLA C, E and G molecules (8). However fetal cells and cell-free DNA can be detected in the maternal blood and trigger T- and B-cell alloimmunization toward paternal HLA antigens (9); evidenced by anti-HLA antibodies in a subset of multiparous women (10–12). In most cases, the maternal immune response is regulated, allowing a healthy pregnancy. The mechanisms leading to the loss of local and systemic immune tolerance during pregnancy are not fully understood but may ultimately result in an alloimmune response toward the fetus, like HLA-mismatched graft rejection after organ transplantation. Data regarding the management of recurrent chronic inflammatory placental disorders are scarce, preventing any definitive recommendations based on the literature.

In this report, we describe a case of RPL caused by CIUE, where comprehensive analysis of cellular and humoral alloimmunity guided a successful treatment approach using immunomodulatory therapies commonly used to prevent allograft rejection in solid organ transplantation. Following this initial experience, a second patient, presenting with similar clinical and histological features, underwent the same treatment protocol and achieved a favorable pregnancy outcome.

## Case report

**Figure 1:**
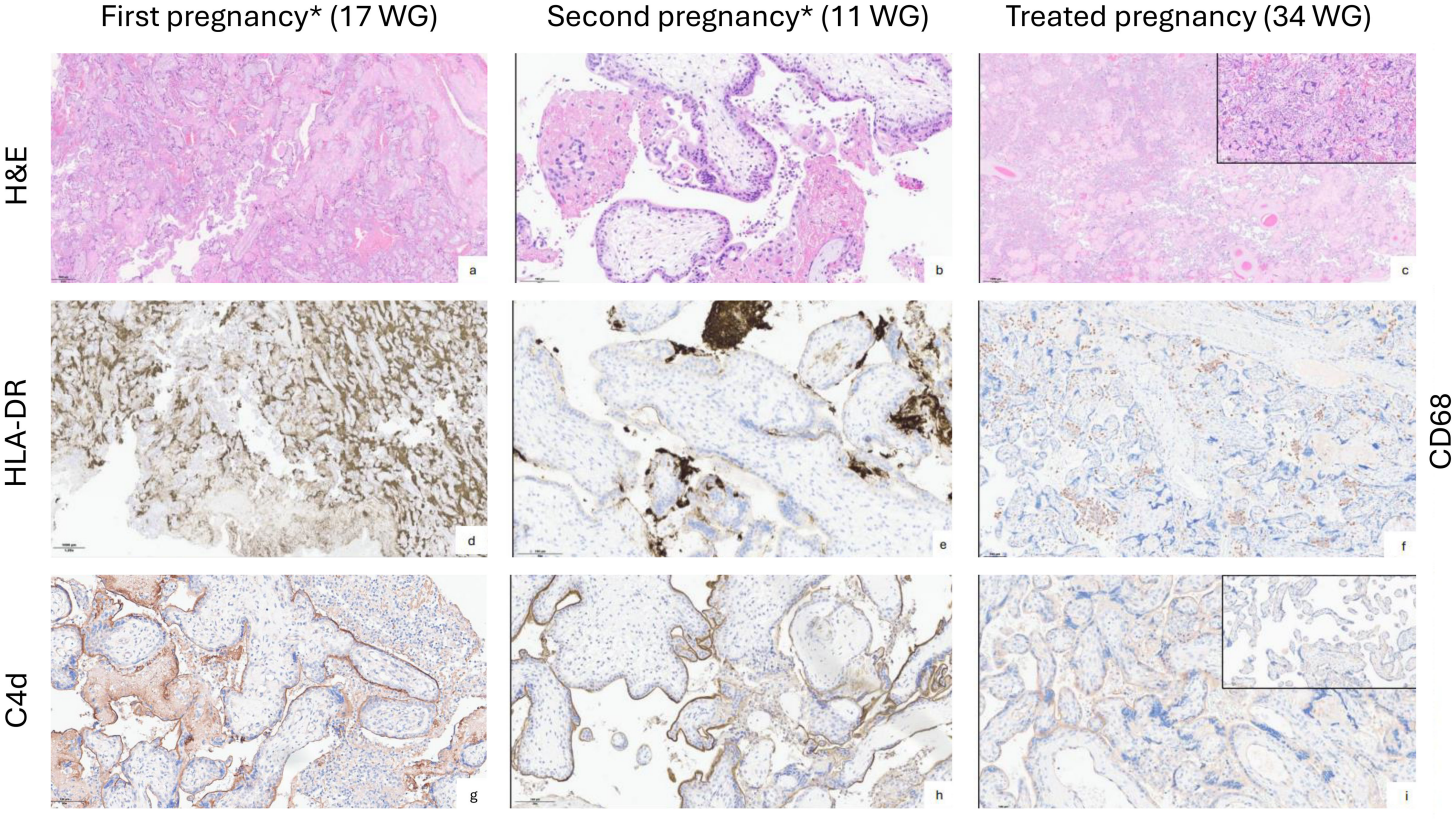
Histological analysis of the placentas comparing untreated and treated pregnancies in the index patient. **(a, d, g)** Pregnancy terminated at 17 WG showing chronic intervillositis (**a**: Hematoxylin and eosin, H&E, x4) with sheets of HLA-DR positive histiocytes filling the intervillous space (**d**: HLA-DR clone CR3/43 monoclonal antibody, Santa Cruz Biotechnology, Inc., x1.25), with strong and diffuse apical C4d positivity of villous trophoblast in the presence of intervillous clusters of mononucleated inflammatory cells (**g**: anti-human C4d antibody (C4dpAb), Biomedica Immunoassays, x20). **(b, e, h)** Second pregnancy marked by miscarriage at 11 WG. Placental histology shows clusters of histiocytes and fibrin deposits in the intervillous space (**b**: H&E, x20), HLA-DR positive intervillous histiocytes and linear apical staining of the villous trophoblast (**e**: HLA-DR immunohistochemical staining, x20), strong and diffuse linear C4d staining of the villous trophoblast (**h**: C4d immunohistochemical staining, x20). **(c, f, i)** Placental parenchyma of the treated current pregnancy showing accelerated villous maturation, increased intervillous fibrin deposition and syncytial knots and foci of chronic intervillositis (inlet) (**c**: H&E, x1.25 and inlet x20), foci of chronic intervillositis with clusters of CD68+ histiocytes (**f**: monoclonal mouse anti-human CD68 (Dako, clone PG-M1), x10), faint linear C4d staining of the villous trophoblast in association with the chronic intervillositis foci (**i**: C4d immunohistochemical staining, x20. Inlet: no significant C4d staining in non-inflammatory areas). All placental examinations were performed by a pathologist specialized in perinatal pathology and with extensive experience in the field. The same person performed retrospective analysis of previous pregnancies material to confirm the findings and chose representative microphotographs for the figures. * The pregnancies were numbered restarting from 1 with the new partner.

A 37-years-old patient had an uneventful first pregnancy with a previous partner, giving birth to a healthy girl at term. She had a history of chronic autoimmune Hashimoto’s thyroiditis, resulting in hypothyroidism treated with levothyroxine 0.125 mg/d, and suffered from allergic rhinitis and atopic dermatitis in childhood. Between 2017-2020, she experienced six successive pregnancy losses with her new partner: the first was a medical termination at 17 + 6/7 weeks of gestation (WG) for severe *in utero* growth retardation, followed by five pregnancy losses between six and nine WG (**Supplementary Table 1**). An in-depth multidisciplinary investigation showed no uterine anomaly on ultrasound, no sexual hormone imbalance, no thrombophilia (including repeated negative antiphospholipid antibodies screening), no active systemic autoimmune disease or inborn errors of immunity. Serologic testing of the mother and histological examination of the placenta ruled out infectious causes. Karyotypes of the couple, as well as on products of conception of the last four pregnancy losses, did not identify any genetic anomaly. When available (pregnancies one, two and three), placental examination showed recurrent CIUE (**Figure 1**). The management of previous pregnancies (summarized in **Supplementary Table 1**) included immunomodulatory agents such as prednisone 10 mg/d, and hydroxychloroquine 200 mg/d, as well as anti-thrombotic drugs (Aspirin 100 mg/d and enoxaparin 40 mg/d), all without success (13, 14).

### Immunological investigations

**Figure 2:**
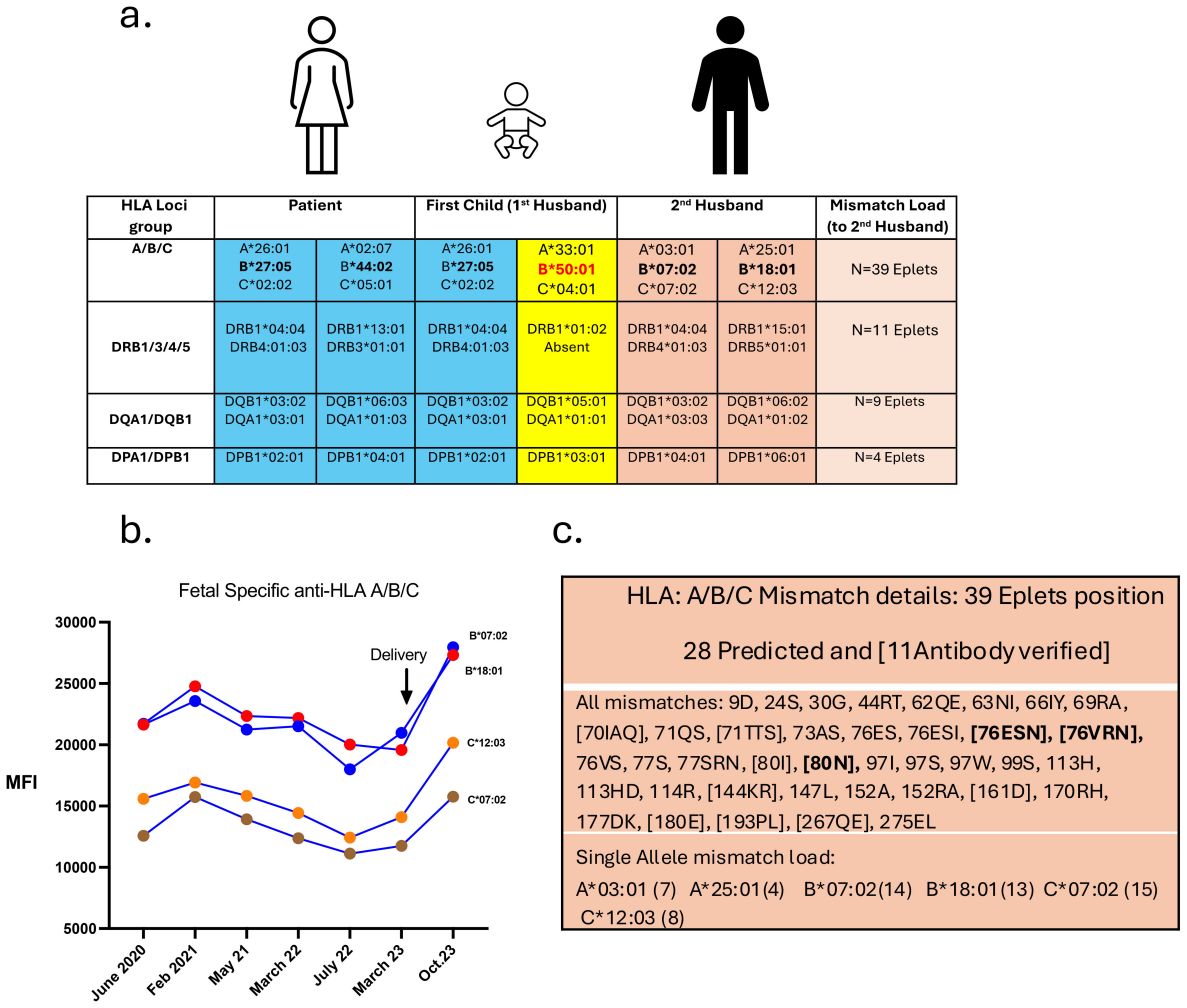
HLA typing of the family and anti-HLA titer evolution throughout the treated pregnancy. **(a)** Table showing HLA genotyping: of the patient (in blue), her first child (first partner with the paternal haplotype shown in yellow), and the genotyping of the second partner (in orange). The calculated mismatch load between the patient and her second partner is depicted on the right column. The high mismatch load on the A/B/C allele is the result of the high HLA-B mismatch in bold: Patient Bw4/Bw4 group, 2nd partner Bw6/Bw6 group. The first pregnancy is acting as a pre-sensitizing event with the 1st partner bearing one HLA-B allele of the Bw6 group, in red. **(b)** Fetal specific anti-HLA Class I antibodies are depicted prior and after delivery. Anti-HLA B*07:02, B*18:01, C*12:03 and C*07:02 are represented, and correspond to the highly probable 80N antibody-verified Eplet (from the historical Bw6 group) after anti-HLA profile analysis. **(c)** Table showing the profile of the Eplet mismatch load between the patient and her second partner. Depicted in bold are the three main Eplets deducted from the antibody profile of the patient: the highly probable 80N Eplet that could be associated to both 76VRN and 76ESN Eplets.

Before considering a new pregnancy, the patient underwent additional investigations. Peripheral blood mononuclear cell subsets, their activation state, and levels of circulating cytokines, chemokines and growth factors were analyzed using multiparameter mass cytometry (CyTOF) and a multiplex bead-based immunoassay. No evidence of immune activation or dysregulation was found, with a normal distribution of T and B-cell subsets including regulatory T cells, and without abnormal baseline systemic inflammatory activity (15). Serum complement proteins levels (C3, C4, CH50, MBL, sC5b-9, factor H, factor Bb) were normal. HLA-typing of the patient and her partner demonstrated numerous high-titter IgG anti-HLA antibodies in the patient’s serum, in particular four with specificity against paternal class I HLA alleles (B7, B18, Cw7, and Cw12; cumulative mean fluorescence intensity, cMFI >40’000) (**Figure 2**). Since the patient had never received transfusions, the alloimmunization was likely due to exposure to paternal-derived fetal HLA antigens during previous pregnancies (16). Using the HLA-EpRegistry, we confirmed a high HLA eplet mismatch load between the second partner and the patient (**Figures 2a–c**). Prior immunization likely occurred during the first pregnancy with the first partner (recurrent Bw6/Bw4 mismatch). As a result, the patient had developed high affinity anti-HLA antibodies corresponding to the highly probable 80N eplet, associated to both 76VRN and 76ESN eplets (**Figure 2c**). We further tested the specificity and pathogenicity of these fetal-specific anti-HLA antibodies (FSA) by performing compatibility tests routinely used in transplantation medicine, i.e., complement-dependent cytotoxicity (CDC) and flow cytometry-based crossmatch tests, using the patient’s serum and her partner’s peripheral blood mononuclear cells (17). The tests were positive for complement-binding class I specific anti-HLA alloantibodies.

### Histological characterization of placentas of previous non-evolutive pregnancies

Histological analysis of the first three pregnancies showed a similar picture of diffuse, high grade intervillositis with the intervillous space being filled by sheets of mononuclear inflammatory cells and increased fibrin deposition, mainly CD68^+^ and HLA-DR^+^ histiocytes with only few intermingled CD8^+^ T cells and CD57^+^ natural killer cells (**Figure 1**). Villous trophoblast cells displayed apical HLA-DR positivity, and strong linear C4d expression (18, 19). Autopsy of the 17-week-old female fetus (1st pregnancy with new partner) showed severe *in utero* growth restriction, short hypomineralized long bones (5^th^-10^th^-percentile), together with signs of chronic hypoxic tissue stress on histology (i.e. renal tubular dysgenesis).

### Therapeutic intervention

Our detailed immunological and histopathological investigations showed convincing evidence of a shared mechanism between CIUE-associated placental dysfunction and solid organ graft rejection, consistent with previous observations (20). We thus selected a regimen that has demonstrated anti-rejection efficacy in highly allosensitized transplant recipients (21), and known to be safe during pregnancy (22), namely intravenous immunoglobulins (IVIG) 2 g/kg every 4-weeks (21, 23, 24), and methylprednisolone 1 mg/kg intravenous bolus at each infusion, to target antibody-mediated placental injuries, combined with tacrolimus (target through levels of 6–8 ng/ml) to control T-cell alloimmune responses and subsequent B-cell activation (**Supplementary Figure 1a**). This therapy, together with hydroxychloroquine and low-dose Aspirin, was introduced 12 weeks prior to a new conception attempt (as in desensitization protocols for solid organ transplantation) and was well tolerated. The treatment was pursued throughout pregnancy, under close monitoring of tacrolimus trough levels and potential side-effects. Parents were extensively counseled about the rationale and possible maternal risks of the proposed therapeutic approach. The risk of fetal demise and the experimental nature of the procedure were also acknowledged. Both parents gave their written informed consent.

### Pregnancy and newborn evolution

The pregnancy was spontaneous. Anatomy ultrasounds and fetal Dopplers at 16 and 20 WG were normal. The patient and fetus were closely monitored throughout the second and third trimesters. Growth ultrasounds and fetal Doppler assessments (including the umbilical arteries, middle cerebral artery, and ductus) were performed every two weeks from 16 to 24 WG. Once fetal viability was reached (24 WG), cardiotocography (CTG) monitoring and biophysical profile (Manning score) were introduced weekly from 24 to 28 WG, increased to twice weekly between 28 and 32 WG, and then three times weekly beyond 32 WG. Serum soluble fms-like-tyrosine-kinase-1 (sFlt-1) to placental-growth-factor (PlGF) ratio (to predict preeclampsia) was used as an early biomarker of placental dysfunction (25, 26). As shown in **Supplementary Figures 1b, c**, a drop in fetal growth curves together with a sharp increase in sFlt-1/PlGF ratio by 20 WG prompted intensification of the immunosuppressive regimen with the introduction of oral prednisolone (10 mg/d) and azathioprine (1 mg/kg/day), together with weekly perfusions of IVIG. Fetal Dopplers remained stable throughout the third trimester. Given the diabetogenic risks of steroids and tacrolimus exposure, and pre-existing overweight (pre-pregnancy BMI 27.3 kg/m^2^), the patient received nutritional counseling. Gestational diabetes was diagnosed at 27 WG and managed through lifestyle modifications without requiring antidiabetic medication. Importantly, the patient experienced no other complications, in particular no pregnancy-induced hypertension or kidney dysfunction, and no infectious complications.

The baby boy was delivered by caesarean section at 33 + 2/7 WG because of non-reassuring fetal monitoring and podalic position. His Apgar scores were 1 (pulse), 10, and 10 at 1, 5 and 10 minutes, respectively. His birth weight was 1300g (percentile-P3), length 40 cm (P5), and head circumference 29.4 cm (P10-25). He required bag mask ventilation for one minute, followed by nasal CPAP for respiratory distress syndrome. The respiratory status improved without the need of surfactant, and respiratory support was weaned off after 48 days. Mild neutropenia (1.8 G/l) and lymphopenia (1.43 G/l) were observed at birth and on day of life (DOL) 4, both normalizing by DOL-25, probably due to azathioprine exposure during pregnancy. Minimal hyponatremia (132 mmol/l) was noted at DOL-4, related to growth restriction and increased demand. Mild kidney dysfunction was noted with peak urea (7.2 mmol/l) and creatinine (91 µmol/l) levels at 36–48 hours of life, normalizing by DOL-4, secondary to pre-birth tacrolimus exposure and its vasoconstrictive effects. The rest of the neonatal course was uneventful. At six months, he showed normal psychomotor development, though he had length growth restriction, with weight catch-up growth. At 2 years of age, the boy is doing well, with appropriate growth parameters and age-appropriate neurodevelopmental milestones.

The placenta was low weight (P< 5) and showed signs of maternal malperfusion, as observed in transplanted pregnant women exposed to calcineurin inhibitors, such as tacrolimus, without a clear delineation of the relationship between placental histopathological findings and tacrolimus exposure (22). Minor foci of CIUE concerning <10% of the placental volume were present (**Figure 1**). Faint apical C4d linear staining of the villous trophoblastic cells was detected only in the areas of intervillous inflammation.

More recently, a second case, a 42-years-old 5G1P patient, without relevant medical history, came to our attention. After a first full term pregnancy, followed by two nonviable intrauterine pregnancies, she suffered a fetal demise at 17 + 3/7 WG during her last pregnancy. Placenta was underweight (P5-P10) with high grade CIUE lesions (**Supplementary Figure 2**). The latter pregnancy loss was attributed to CIUE, as histological evidences were clear and alternative causes could be excluded. She had anti-HLA class I (A2, B8) FSA and no clinical or biological sign of autoimmunity. Already pregnant (6 WG) at her first visit, we immediately began therapy after obtaining parental written consent, with intensive induction for four weeks followed by a maintenance phase, with similar maternal and fetal surveillances (**Supplementary Figure 2**). The pregnancy went uneventful, except for gestational diabetes diagnosed at 27 WG and managed with insulin. Biological monitoring showed normal serum sFlt-1/PlGF ratio and harmonious fetal growth on ultrasounds. She delivered via elective caesarean section for *vasa previa* at 36 WG. As extraction was prolonged, the baby boy suffered from multiple bruising and initially acute respiratory distress. His arterial blood pH was 7.19 and lactate 4.2 mmol/L. His Apgar scores were 0, 7 (-1 point for reactivity, and -2 for tonus) and 10 at 1, 5 and 10 minutes respectively. Birth weight, height and cranial perimeter were all in normal range for gestational age. The evolution was rapidly favorable, and he could be discharged home on day 9. Placenta histology showed no sign of villitis or intervillitis on standard staining and immunohistochemistry (HLA-DR); nor C4d staining in the trophoblastic villous lining (**Supplementary Figure 2**).

## Discussion

**Figure 3:**
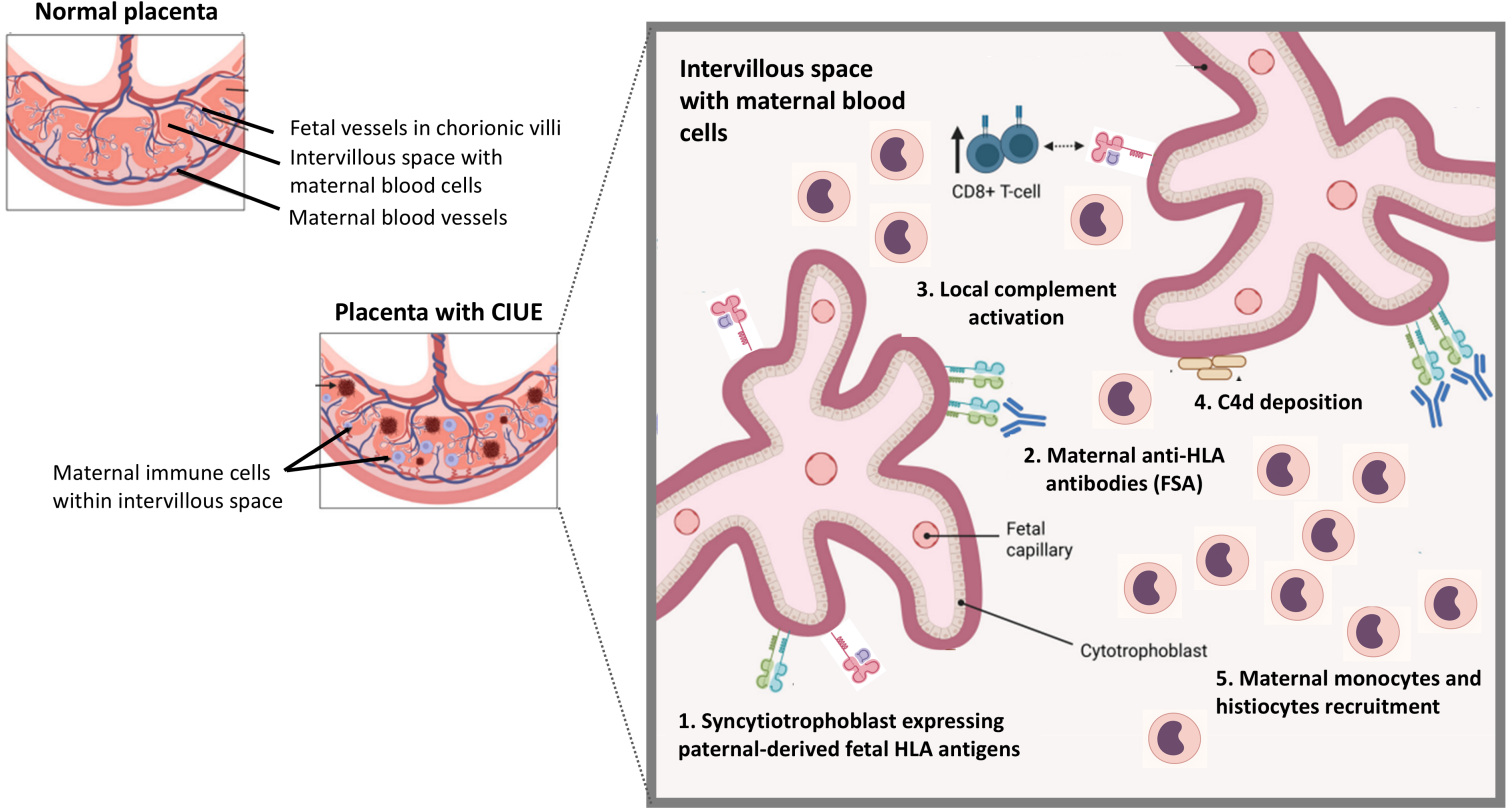
Schematic representation of chronic intervillositis of unknown etiology (CIUE). Left: comparison of a normal placenta and a placenta with CIUE, characterized by maternal immune cells infiltration of the intervillous (maternal) space. Right: proposed immunopathological cascade. (1) Syncytiotrophoblast expressing paternal-derived fetal HLA molecules (fetal antigens). (2) Maternal anti-HLA antibodies (fetal-specific anti-HLA antibodies, FSA) bind to trophoblast HLA molecules. (3) Complement activation ensues. (4) C4d deposition is observed at the trophoblast surface. (5) Recruitment of maternal monocytes and histiocytes amplifies inflammation within the intervillous space. This process mirrors antibody-mediated rejection in solid organ transplantation. Figure adapted from (33).

These cases reinforce the concept of CIUE as a pathological state of loss of immunological tolerance toward paternal antigens, leading to placental injury comparable to allograft rejection after solid organ transplantation (27). Consistent with the role of alloimmunity, severity increases with subsequent pregnancies and re-exposure to paternal HLA. CIUE recapitulates the immunological and histological hallmarks of active antibody-mediated allograft rejection (28). Hence, the main culprits are maternal fetal-specific anti-HLA antibodies (FSA) rather than effector T cells. The presence of the terminal complement split product C4d (or C3d) with linear deposition on villous trophoblast cells (**Figure 1**) further illustrates the pathogenic role of maternal FSA. By binding to fetal endothelial cells, these antibodies activate the complement classical pathway locally, at the maternal-fetal interface, with subsequent inflammatory and ischemic tissue injury and placental dysfunction, as suggested by others (**Figure 3**) (29). This emphasizes the added diagnostic value of histopathology in RPL cases.

In this investigational work, the pregnancy outcomes of two consecutive patients were positive, however this work suffers some limitations warranting further exploration, ideally within a proper clinical trial. The first and most import limitation is the small number of cases and their retrospective report. Second, CIUE was present on histology analysis from previous pregnancy losses, and most alternative causes could be ruled out, however karyotypic analysis could not be performed on all previous pregnancy losses, due to the absence of material. Finally, the presence of anti-HLA antibodies is a frequent finding among women and correlates with the number of pregnancies (11, 12, 30). However, only a minority are clinically significant (31). In the present report, the results of the complement-dependent cytotoxicity (CDC) and flow cytometry-based crossmatch tests, using the patient’s serum and her partner’s peripheral blood mononuclear cells, demonstrate the specificity and pathogenicity of these antibodies against fetal tissues.

Overall, these findings underscore the relevance of anti-HLA alloantibodies as a diagnostic biomarker and therapeutic target in at-risk pregnancies. Various immunomodulatory therapies have previously been attempted in CIUE, targeting either innate immune cells, T-cell activation, inflammatory cytokines or the coagulation pathway, with inconsistent results (32). As our patients presented the clinical and histopathological criteria of antibody-mediated anti-fetal immune responses, we implemented an immunosuppressive protocol derived from transplantation medicine that led to successful pregnancy outcomes. This approach combined immunomodulation with IVIG, tacrolimus, steroids and hydroxychloroquine, along with close monitoring of immunological parameters and placental function, ultimately resulting in two live births after several CIUE-related pregnancies losses. Furthermore, in the first case, treatment intensification was able to rescue early placental insufficiency as demonstrated by subsequent normalization of the serum sFlt-1/PlGF ratio and further fetal growth. Furthermore, while we combined several agents commonly used in antibody-mediated rejection, not all may be required in every patient; future studies are needed to define the minimal effective regimen and assess safety.

These cases contribute to the growing evidence that alloimmunity may be central to the pathogenesis of CIUE and suggest that therapies targeting antibody-mediated immune responses could offer new perspectives for patients with recurrent pregnancy losses linked to this condition. Further studies are required to delineate optimal treatment regimens and identify predictive markers of response to immunomodulatory therapy.

## Data Availability

The original contributions presented in the study are included in the article/**Supplementary Material**. Further inquiries can be directed to the corresponding author.

## Supplementary material

Supplementary Table 1 Summary of patient’s obstetric history. The table below details all previous pregnancies of the index patient with her new partner, describing year of conception, therapy received for the pregnancy, term of the pregnancy loss as well as results of placental and fetal material analysis (when available). WG, weeks of gestation; NA, not available; mg/d, milligram per day. NB: The first uneventful pregnancy, with a different partner, took place in 2011.

Supplementary Figure 1 Summary of the therapy phases and biological surveillance of the index pregnancy. **(A)** Summary of treatment protocol and surveillances. **(B)** Evolution of serum sFlt-1/PlGF ratio during pregnancy. **(C)** Fetal and neonatal growth. The vertical arrows indicate the time-point of protocol intensification.

Supplementary Figure 2 Therapy, biological surveillance and representative placental histology from the second case. Placenta from her previous pregnancy marked by fetal demise at 17+ WG was underweight (P5-P10) **(a, c, e)** with histology showing high-grade chronic intervillositis (**a**: Hematoxylin and eosin, x10) diffuse intervillositis and increased intervillous fibrin deposition (**c**: HLA-DR immunostaining, 5x) as well as clusters of intervillous histiocytes surrounding the C4d-positive chorionic villi (**e**: C4d immunohistochemical staining, x20). As she was already at 6 WG at her first visit in our center, therapy was begun immediately using a slightly modified treatment protocol with intensive induction for 4 weeks followed by a maintenance phase, with similar maternal and fetal surveillances **(g)**. The pregnancy went uneventful, with biological monitoring showing normal serum sFlt-1/PlGF ratio and harmonious fetal growth on ultrasounds **(h)**. Placenta histology **(b, d, f)** showed no sign of villitis or intervillitis on standard staining (1b: Hematoxylin and eosin, x10) and by immunohistochemistry (**d**: HLA-DR immunostaining, 10x); no C4d staining in the trophoblastic villous lining (**f**: C4d immunostaining, 20x).
