# Supplementary material for: Case Report: A novel approach to prevent chronic histiocytic intervillositis and recurrent pregnancy loss by targeting maternal alloimmunity
Source: Front Immunol. 2025 Nov 10;16:1693016. doi: 10.3389/fimmu.2025.1693016 (PMC12640819; doi:10.3389/fimmu.2025.1693016)

***Supplementary Material***

**Case report: a novel approach to prevent chronic histiocytic intervillositis and recurrent pregnancy loss by targeting maternal alloimmunity**

**Mathilde Gavillet^1,2^, Carole Gengler^3^, Helene Legardeur^4^, Monique Gannagé^5^, Jardena Puder^4^, Lydie Beauport^4^, Alice Panchaud^4,6,7^, Samuel Rotman^3^, Denis Comte^8^, David Baud^4^*, and Dela Golshayan^9^***

1) Service of Hematology, Department of Oncology, Lausanne University Hospital and University of Lausanne, Switzerland

2) Central Laboratory of Hematology, Department of Laboratory Medicine and Pathology, Lausanne University Hospital, Switzerland

3) Institute of Pathology, Department of Laboratory Medicine and Pathology, Lausanne University Hospital and University of Lausanne, Switzerland

4) Woman-Mother-Child Department, Lausanne University Hospital and University of Lausanne, Switzerland

5) Service of Immunology and Allergy, Lausanne University Hospital, Switzerland,

6) Service of Pharmacy, Lausanne University Hospital and University of Lausanne, Switzerland,

7) Institute of Primary Health Care (BIHAM), University of Bern, Switzerland,

8) Division of Internal Medicine, Department of Medicine, Lausanne University Hospital and University of Lausanne, Switzerland,

9) Transplantation Centre and Transplantation Immunopathology Laboratory, Department of Medicine, Lausanne University Hospital and University of Lausanne, Switzerland

* Similar contribution

**Supplementary Table 1. Summary of patient’s obstetric history.**

The table below details all previous pregnancies of the index patient with her new partner, describing year of conception, therapy received for the pregnancy, term at pregnancy loss as well as results of placental and fetal material analysis (when available).

| Year of conception | Trialed medication | Week of gestation at pregnancy loss | Placenta/fetal tissue analysis after pregnancy loss |
| --- | --- | --- | --- |
| 2017 | None | 17+6/7 | Early intrauterine fetal growth restriction. Massive chronic intervillositis with increase in peri-villous fibrin deposition. |
| 2018 | None | 11+6/7 | Massive chronic intervillositis with increase in peri-villous fibrin deposition. |
| 2018 | *From pregnancy diagnosis onward*  Aspirin 100 mg/d  Hydroxychloroquine 200 mg/d  Prednisone 10 mg/d | 6+3/7 | Very little materiel, showing intervillous cellular deposition and highly positive for HLA-DR staining, indicative of immune-mediated pathogenesis. |
| 2019 | *Preconceptionally*  Aspirin 100 mg/d  Hydroxychloroquine 200 mg/d  Prednisone 20 mg/d  *Additional from pregnancy diagnosis (6 WG) onward*  Pravastatin 10 mg/d  Enoxaparin 40 mg/d | 8+3/7 | NA |
| 2019 | *Preconceptionally*  Aspirin 100 mg/d  Hydroxychloroquine 400 mg/d  Prednisone 20 mg/d  *Additional from pregnancy diagnosis (6 WG) onward*  Pravastatin 10 mg/d  Enoxaparin 40 mg/d | 7+2/7 | NA |
| 2020 | *From pregnancy diagnosis (6 WG) onward*  Aspirin 100 mg/d  Prednisone 5 mg/d  Tacrolimus (through level 6-8 ng/ml) | 8 | NA |

WG: weeks of gestation; NA: not available; mg/d: milligram per day

NB: The first uneventful pregnancy, with a different partner, took place in 2011.

**Supplementary Figure 1. Summary of the therapy phases and biological surveillance of the index pregnancy.**

**Panel A.** Summary of treatment protocol and surveillances. **Panel B.** Evolution of serum sFlt-1/PlGF ratio during pregnancy. **Panel C.** Fetal and neonatal growth. The vertical arrows indicate the time-point of protocol intensification.


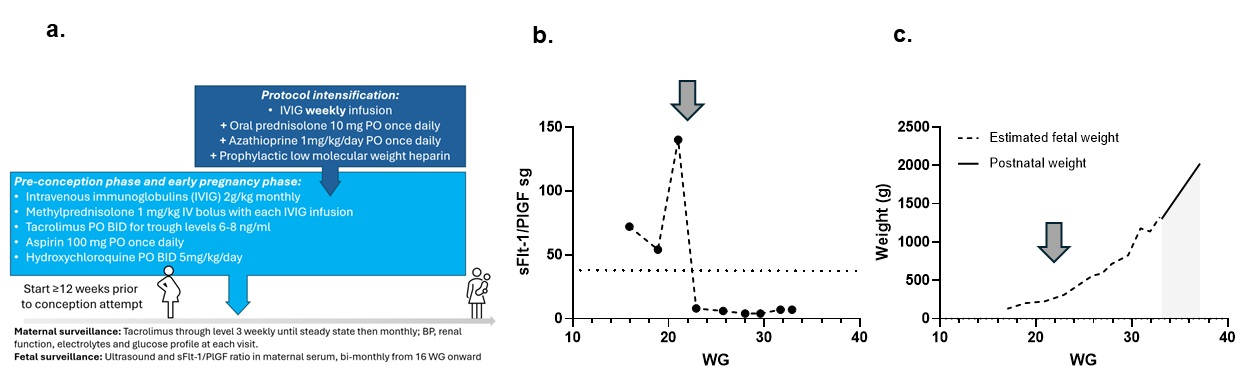


**Supplementary Figure 2. Therapy, biological surveillance and representative placental histology from the second case.**

Placenta from her previous pregnancy marked by fetal demise at 17+* WG) was underweight (P5-P10) (**Panel a, c, and e)** with histology showing high-grade chronic intervillositis (**1a:** Hematoxylin and eosin, x10) diffuse intervillositis and increased intervillous fibrin deposition (**1c:** HLA-DR immunostaining, 5x) as well as clusters of intervillous histiocytes surrounding the C4d-positive chorionic villi (**1e:** C4d immunohistochemical staining, x20). As she was already at 6 WG at her first visit in our center, therapy was begun immediately using a slightly modified treatment protocol with intensive induction for 4 weeks followed by a maintenance phase, with similar maternal and fetal surveillances (**Panel g)**. The pregnancy went uneventful, with biological monitoring showing normal serum sFlt-1/PlGF ratio and harmonious fetal growth on ultrasounds (**Panel h**). Placenta histology (**Panel b, d, and f)** showed no sign of villitis or intervillitis on standard staining (**1b:** Hematoxylin and eosin, x10) and by immunohistochemistry (**1d:** HLA-DR immunostaining, 10x); no C4d staining in the trophoblastic villous lining (**1f:** C4d immunostaining, 20x).


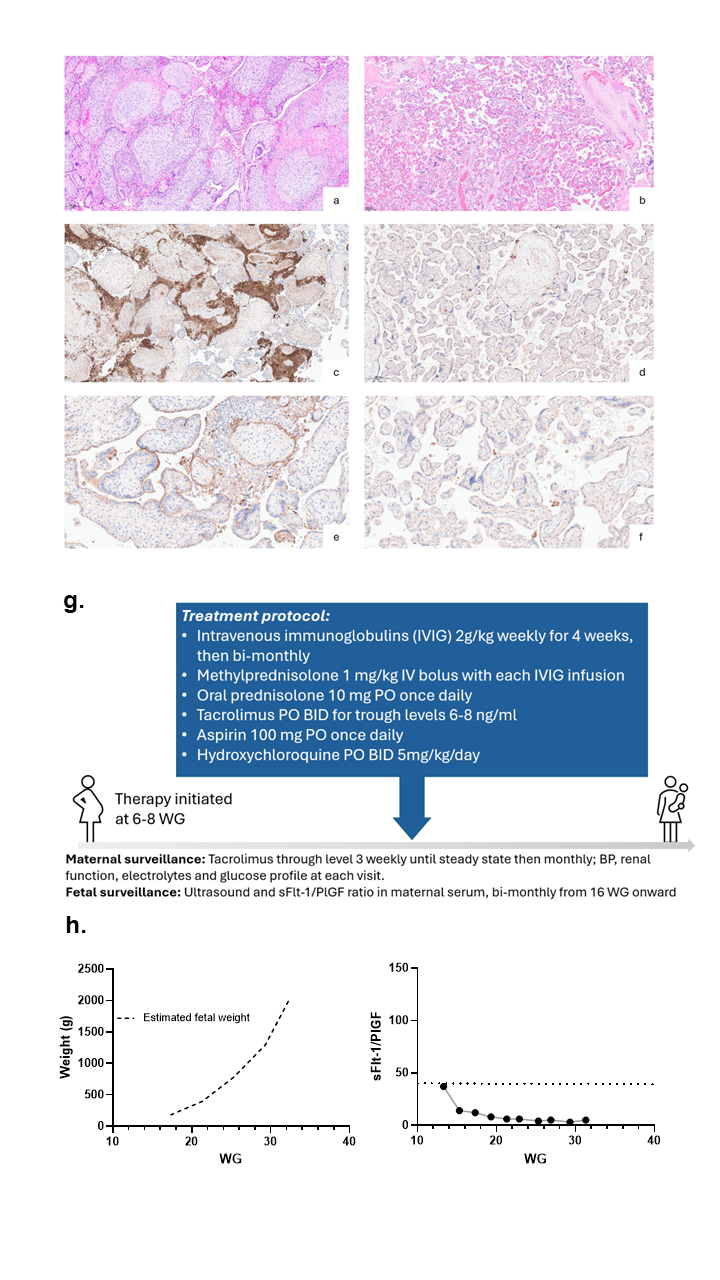

Supplement: Supplementary Table 1 — Summary of patient’s obstetric history. The table below details all previous pregnancies of the index patient with her new partner, describing year of conception, therapy received for the pregnancy, term of the pregnancy loss as well as results of placental and fetal material analysis (when available). WG, weeks of gestation; NA, not available; mg/d, milligram per day. NB: The first uneventful pregnancy, with a different partner, took place in 2011. [file DataSheet1.docx]
